# The impact of education programs on smoking prevention: a randomized controlled trial among 11 to 14 year olds in Aceh, Indonesia

**DOI:** 10.1186/1471-2458-13-367

**Published:** 2013-04-19

**Authors:** Teuku Tahlil, Richard J Woodman, John Coveney, Paul R Ward

**Affiliations:** 1 Discipline of Public Health, School of Medicine, Flinders University, Adelaide, SA 5001, Australia; 2 Discipline of General Practice, School of Medicine, Flinders University, Adelaide, SA 5001, Australia; 3 Nursing Department, Medicine Faculty, Syiah Kuala University, Banda Aceh 23111, Indonesia

**Keywords:** Smoking prevention, Indonesia, Schools, Health knowledge, Attitude, Behavior, Intention

## Abstract

**Background:**

School-based smoking prevention programs have been shown to increase knowledge of the negative effects of smoking and prevent tobacco smoking. The majority of evidence on effectiveness comes from Western countries. This study investigated the impact of school-based smoking prevention programs on adolescents’ smoking knowledge, attitude, intentions and behaviors (KAIB) in Aceh, Indonesia.

**Methods:**

We conducted a 2 × 2 factorial randomized controlled trial among 7^th^ and 8^th^ grade students aged 11 to 14 years. Eight schools were randomly assigned to a control group or one of three school-based programs: health-based, Islamic-based, or a combined program. Students in the intervention groups received eight classroom sessions on smoking prevention education over two months. The KAIB impact of the program was measured by questionnaires administered one week before and one week after the intervention.

**Results:**

A total of 477 students participated (58% female, 51% eighth graders). Following the intervention, there was a significant main effect of the Health based intervention for health knowledge scores (β = 3.9 ± 0.6, p < 0.001). There were significant main effects of the Islamic-based intervention in both health knowledge (β = 3.8 ± 0.6, p < 0.001) and Islamic knowledge (β = 3.5 ± 0.5, p < 0.001); an improvement in smoking attitude (β = −7.1 ± 1.5, p < 0.001). The effects of Health and Islam were less than additive for the health and Islamic factors for health knowledge (β = −3.5 ± 0.9, p < 0.01 for interaction) and Islamic knowledge (β = −2.0 ± 0.8, p = 0.02 for interaction). There were no significant effects on the odds of intention to smoke or smoking behaviors.

**Conclusions:**

Both Health and Islamic school-based smoking prevention programs provided positive effects on health and Islamic related knowledge respectively among adolescents in Indonesia. Tailoring program interventions with participants’ religion background information may provide additional benefits to health only focused interventions.

**Trial registration:**

Australia and New Zealand Clinical Trials Register, ACTRN12612001070820

## Background

Tobacco smoking is a widespread phenomenon and an accepted cultural habit for many young Indonesians [1]. Indonesia is ranked as the world’s third highest tobacco smoking nation [2]. In 2006, a national survey of 3,737 students aged 13 to 15 years showed that 37.7% had smoked cigarettes, 13.5% were identified as current tobacco smokers, 11.8% were current cigarette smokers, and 3.8% reported being current users of other tobacco products [3]. It was also reported that 95.1% of the Indonesians adolescents who reported never smoking had expressed their intention to start smoking in the next 12 months [3]. At a provincial level, 29.7% of adolescents over 10 years old are active smokers in Aceh Province [4]. The level of cigarette smoking among smokers in Aceh (19 cigarettes per day) is also higher than the average national rate (12 cigarettes per day) [4].

A comprehensive tobacco control program requires a combination of educational, clinical, regulatory, economic, and social strategies [5]. Unfortunately, tobacco control regulations have not been strictly imposed in Indonesia [2]. Moreover, Indonesia is the only country in South-East Asia and one of the few countries in the world that has not signed the World Health Organization Framework Convention on Tobacco Control [FCTC] [2,6]. Thus, increasing people’s knowledge about the adverse health effects of tobacco use is currently considered the most appropriate and effective strategy for tobacco control in Indonesia [6].

School-based smoking prevention programs are considered to be one of the most effective strategies for reducing smoking prevalence among adolescents in general [7]. Such programs have been shown to improve adolescents’ smoking knowledge [8,9] and attitude [8,9], and reduce smoking intention [9-11] and behaviors [10,12]. However, school-based smoking prevention programs may differ in many respects, including the target participants, study design, type of intervention, intensity, and measured outcomes [8,13,14]. In addition, the vast majority of programs undertaken to date have been in developed countries and therefore little is known about the effectiveness of such programs in developing countries [15]. In particular, to our knowledge there is no study that has assessed the implementation of smoking prevention or cessation programs within schools in Indonesia.

There is evidence that religiosity/spirituality can have positive effects on adolescents’ health attitude and behaviors, including tobacco smoking [16]. Religiosity reduced the risk of tobacco smoking and other risky behaviors because strong religious perception and attendance at worship services were associated with reduced risk of smoking [17]. However, there is currently no agreement among experts about the effectiveness of Islamic teaching in tobacco smoking prevention and cessation programs. The reviewed literature suggests that we lack an evidence base for this approach [18].

Based on the above evidence, we designed a study to evaluate three interventions. The first, the health-based intervention, drew on best-practice models from the literature [9-12,19,20]; the second, the Islamic-based intervention, was specifically developed for use in Muslim contexts and included Islamic teaching [21,22] related to tobacco smoking; while the third intervention, the combined intervention, included key aspects of the other two interventions. We investigated the impact of these school-based smoking prevention programs on adolescents’ smoking Knowledge, Attitude, Intention to smoke and subsequent smoking Behavior (KAIB) in schools in Aceh Province, Indonesia. The KAIB variables are frequently measured [9,23] in school-based smoking prevention programs.

## Methods

### Participants

Participants were recruited using a combination of convenience and simple random sampling [10]. With the assistance of the District Head of the Education Department, we invited junior high schools in a district of Aceh Province, Indonesia, to take part in the study. From those schools where school principals agreed to participate, we selected students aged between 11 and 14 years from 7^th^ and 8^th^ grades in eight junior high schools. The schools were located in the same area and geographically close to each other, with a distance of about 4 km from one to another. There were no differences between schools in term of students’ size, gender, religion, and race/ethnic composition.

#### Randomization

**Figure 1:**
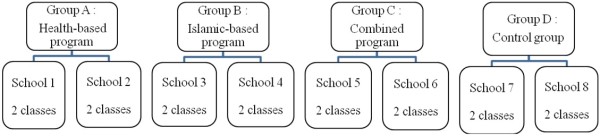
Study participants by intervention approach.

The eight selected schools were randomly assigned using block randomization to a control group (2 schools) or one of the three smoking prevention interventions (2 schools for each intervention) (Figure 1).

### Program development

Prior to the development of the program, six face-to-face interviews and ten telephone interviews were conducted with junior high school teachers, former junior high school teachers, and staff from the Department of Education in Aceh Province, Indonesia. These in-depth interviews were aimed at identifying how to provide smoking prevention education programs that were culturally, politically, and pedagogically appropriate for Acehnese adolescents in particular and young Indonesians in general. Interviewees were asked about their opinions and perceptions about the need for a student program, the scope and required components of the program, the optimal type of program providers, and possible barriers to implementation of school-based smoking prevention education programs in Aceh.

The findings of this investigation highlighted several key recommendations that served as the basis for the development of our program interventions. These included: (1) that school teachers and policy makers firmly supported implementation of school-based smoking prevention education programs in Aceh; (2) the inclusion of both health- and Islamic-based components into such programs was highly recommended and considered a culturally appropriate approach for adolescents in Aceh; and (3) the program should involve school teachers and experts from relevant areas, and be integrated into existing relevant school subjects during school hours.

### Program intervention and implementation

1. The health-based intervention

The health-based intervention consisted of delivering health-based smoking prevention knowledge and skills to the students. The program curriculum consisted of eight two-hour classroom sessions, over eight weeks, addressing the following areas: tobacco smoking, the prevalence and incidence of tobacco smoking in Indonesia, the adverse effects of smoking, smoking laws in Indonesia, and cigarette refusal techniques including stress management. Teaching methods included lectures, demonstrations, and active learning activities such as the use of discussions, role playing, playing games/sport, and storytelling.

2. The Islamic-based intervention

Students who participated in this program learned and practiced smoking prevention skills based on Islamic teaching. This intervention included eight two-hour classroom sessions focusing on the basic concepts of Islam, health concepts in Islam, smoking behavior in Islamic society, Islamic law concerning smoking, the dangers of smoking, and healthy living techniques without smoking in Islam. Teaching methods were similar to the health-based intervention.

3. The combined intervention

This intervention included components from the other two interventions and comprised eight two-hour classroom sessions. It included the concept of Islam as a religion, health concepts in Islam, tobacco smoking behaviors in Indonesia, the adverse effects of smoking, smoking rules in Indonesia from a national and an Islamic view, healthy living techniques without smoking in Islam, and cigarette refusal techniques including stress management. Students learned and practiced their skills through class activities similar to the other two interventions.

All programs were delivered to students in their usual classroom setting, during school hours, combined with relevant school subjects such as Islamic teaching, biology, civic education, physical education, and local content courses.

Participants in the control schools did not receive any smoking prevention education program but were tested (using the KAIB instrument) at the beginning and the end of the study.

### Program providers

Providers (teachers) were selected by the researchers in collaboration with school principals and/or school coordinators. For the health-based program, providers included school teachers, health educators, and health professionals; for the Islamic-based program, providers included Islamic leaders and school teachers with in-depth knowledge of Islamic teaching about smoking prevention; and for the combined program, program providers included health professionals and educators, and religious leaders or school teachers with in-depth knowledge of Islamic teaching in relation to smoking prevention.

A one-day training session for the program providers was designed to maximize the success of the program implementation. This training also meant that each intervention was delivered similarly, irrespective of school or provider. Additionally, the training was aimed at gaining support for the program from policy makers, school administrators, teachers, and the community. Training activities included an introduction to the program, an overview of suggested teaching activities and methods, and a review of available resources. Both the researchers and experts from the health and educational fields were involved in the training to share their expertise and experience with providers.

### Ethical approval

The study was approved by the Social Behavioral Research Ethic Committee (SBREC) of Flinders University. Written informed consent was obtained from students, their parents or guardians, and the school principals. Student participation was voluntary and students could withdraw from the study at any time.

### Primary outcomes

The primary outcomes of the study were smoking knowledge, attitude, intentions, and behaviors, which were assessed using a questionnaire that was adapted from previous studies [10,12,24] and tailored towards the educational material to be delivered in the three intervention programs.

### Development and testing of instrument

The questionnaire was developed in English and translated into Indonesian. To ensure that the questionnaire was suitable for junior high school students in Aceh, we invited six teachers from the selected schools to provide their comment on the Indonesian version of the questionnaire. No revision was needed following questionnaire evaluation. The questionnaire was pilot tested with 70 students in 7^th^ and 8^th^ grades at one junior high school in Aceh, Indonesia. Test–retest reliability was measured in a regular classroom setting with a 2-week interval between Test 1 and Test 2 [25].

#### Demographic information

Information was collected on sex, age (in years), year or class of study (7^th^ or 8^th^ grade), and current living condition (living with both parents, with one parent and stepfather/mother, with one parent only, with relatives, or with others).

#### Health knowledge

The health knowledge scale comprised 20 questions related to smoking prevalence in Indonesia, national regulation of tobacco control in Indonesia, and harmful effects of cigarette smoking. Each question was presented in a multiple choice format with four possible options for each answer, with one point awarded for each correct answer. The total score for this scale ranged from 0 to 20, with higher scores representing greater knowledge.

Initially, a 50-item questionnaire with true/false responses was developed using program materials and questions from previous studies [26-28]. However, pilot testing showed low test–retest reliability and modifications were made: (1) removal of questions (25 items) with low internal consistency and reliability or those that appeared to be too easy, (2) addition of new questions that were more closely related to the program materials, and (3) transforming the questions into a multiple choice format. Cronbach’s alpha (α) for the modified scale was 0.88 (Test 1) and 0.90 (Test 2), whereas the kappa measure of agreement ranged from 0.08 to 0.53.

#### Islamic knowledge

The Islamic knowledge scale comprised 20 questions on Islamic teaching and rulings on cigarette smoking. Each question was presented in a multiple choice format with four possible options for each answer, with one point awarded for each correct answer. The total score for this scale ranged from 0 to 20, with higher scores representing greater knowledge.

Initially, 30 multiple choice questions were prepared based on the information in the program materials [21,22,29-31] each with four alternative responses. However, following pilot testing 10 items were removed because of low internal consistency and content validity (α) (Test 1 = 0.65, Test 2 = 0.80). Internal consistency coefficients (α) for the shorter scale increased to 0.79 (Test 1) and 0.88 (Test 2), whereas the kappa measure of agreement ranged between 0.02 and 0.66.

#### Smoking attitude

Attitude to smoking was evaluated using 25 statements derived and modified from previous studies [12,20,28,32-34]. Statements were presented in a five-point Likert scale format, with responses ranging from 0 (strongly disagree) to 4 (strongly agree) for positively worded items, and 0 (strongly agree) to 4 (strongly disagree) for negatively worded items. Scores were summed to obtain a total score for smoking attitude, which ranged from 0 to 100. A higher score indicated that the individual was more likely to smoke.

Initially a 50-item scale was prepared, with statements focused on participants’ attitudes towards a range of positive and negative perceptions [35] about cigarette smoking, including its physical consequences (e.g. cigarette smoking is harmful to your health), addiction (e.g. a person could easily get addicted to smoking), smoking policy (e.g. cigarette companies should not be allowed to advertise by sponsoring athletic events), economic effects (e.g. smoking is a waste of money), and the social effects and benefits of smoking (e.g. smoking enhances popularity and social bonding). From these 50 items, 25 were selected for the study following pilot testing for test–retest reliability. Reasons for deleting items included low internal consistency coefficients (α), theoretical or practical reasons [36]. Internal consistency coefficients (α) for the selected 25 items were 0.87 (Time 1) and 0.86 (Time 2), with the kappa values agreement ranged between 0.02 and 0.52.

#### Smoking intention

Smoking intention was assessed using three questions each with five response categories, ranging from 0 for ‘certain not to smoke’ to 4 for ‘certain to smoke’. The questions were adapted from previous studies and asked participants whether they would smoke tobacco (cigarettes) next year [10,33], during senior high school [9], when older, or when over 50 years of age [37]. Because there were very low responses to the higher categories, the five categories were collapsed into a dichotomous variable: ‘no intention to smoke’ (combining ‘certain not to smoke’ and ‘very unlikely to smoke’) and ‘intention to smoke’ (combining ‘undecided’, ‘likely to smoke, or certain to smoke). We classified ‘undecided’ as ‘intending to smoke’ because such respondents have a higher risk for smoking [37].

#### Smoking behavior

Smoking behavior was assessed using three questions: the number of cigarettes smoked in the last seven days [10,38], in the last 30 days [10,12,20,24,28,38], and in the subject’s lifetime [20]. To assess cigarette smoking frequency in the last seven days, response categories were ‘never tried a cigarette, not even one puff’ (0), ‘one puff or two puffs’ (1), ‘just one cigarette’ (2) ‘two cigarettes’ (3), and ‘three to five cigarettes’ (4). For assessing the frequency of cigarettes smoked in the last month, we used the following response categories: ‘I did not smoke cigarettes during the past 30 days’ (0), ‘less than one cigarette per day’ (1), ‘one cigarette per day’ (2), ‘two to five cigarettes per day’ (3), ‘six to ten cigarettes per day’ (4), ‘11 to 20 cigarettes per day’ (5), ‘more than 20 cigarettes per day’ (6) [24,28]. Finally, for assessing frequency of lifetime cigarette smoking, the responses included ‘I did not smoke any cigarettes’ (0), ‘less than one cigarette’ (1), ‘one cigarette’ (2), ‘two to five cigarettes’ (3), ‘six to ten cigarettes’ (4), ‘11 to 20 cigarettes’ (5), ‘21 to 60 cigarettes’ (6), ‘61 to 100 cigarettes’ (7), and ‘more than 100 cigarettes’ (8). Because the expected frequencies in certain cells were less than 5, for the analysis we collapsed these categorical responses into two groups: 0 = never smoked and 1 = smoker, at least one puff or above.

### Study procedure

Pre and posttest questionnaires were administered one week before and one week after the program intervention, respectively [9]. Tests were administered in the students’ usual classroom by research assistants and program providers under the supervision of the researchers who were not affiliated with the schools.

### Statistical analysis

Sample size was determined according to results of a pilot study that allowed an estimate of the mean and standard deviation (SD) for health knowledge, which was the primary outcome of the study. Ignoring the effects of clustering within schools which was anticipated to be small, we calculated that we would need to enroll 480 students for the study to have 80% power to show an absolute between-group difference in the primary outcome measure at a 2-sided alpha level of 0.05, assuming a 3-point improvement in health knowledge and a SD for the health knowledge score of 6.62.

Differences between groups at baseline in subject characteristics, and each of the primary outcomes (knowledge, attitude, intention, and behavior) were assessed using chi-squared tests of association and ANOVA as appropriate. The impact of the interventions on the primary outcomes was assessed using generalized linear models with adjustment for baseline scores and the use of robust standard errors to account for clustering within each classroom. In our models we used health outcomes as the dependent variable, and the treatment factors as independent variables. Specifically, we included a main effects term for each of the two interventions (health and Islam) and assessed the presence of an interaction between the two using a Health × Islam interaction term if either of the main effects for health Islam were significant. We also assessed the degree of clustering within classrooms for each outcome by using a random intercept model with the variable of time (pre or posttest) as an additional covariate and subject identifier as the random effect. The effects of clustering were then reported as an intra-class correlation coefficient (ICC).

A Type 1 error rate of p < 0.05 was used as the standard criterion for judging the statistical significance of main effects and interactions in each analysis. All analysis was completed using Stata version 12.0 (StataCorp, Texas, USA).

## Results

### Subject characteristics

A total of 477 students participated in the study, with 476 students completing the questionnaire at pretest (one student absent) and 477 students at posttest. The analyzed data comprised participants who completed pre and posttests (n = 476). Of these students, 128 (27%) were assigned to the control group, 122 (26%) to the health-based program, 109 (23%) to the Islamic-based program, and 109 (25%) to the combined program. Table 1 describes the characteristics of the participants according to the study groups. More than half of the students were female (58%), in 8^th^ grade (51%), and aged over 12 years (67%) at baseline. Most of the students lived with their parents (84%). No significant differences were noted between groups with regard to sex, age, year of study/grades, and current living conditions.

**Table 1:** Characteristics of participants

**Characteristics**	**Health (n = 122)**	**Islamic (n = 109)**	**Combined (n = 117)**	**Control (n = 128)**	**p values**^**1**^
Sex					0.92
Boys (%)	42.6	42.2	38.5	41.4	
Girls (%)	57.4	57.8	61.5	58.6	
Age					
11 years (%)	1.6	1.8	2.6	0.8	0.50
12 years (%)	23.0	32.1	31.6	38.3	
13 years (%)	48.4	45.9	42.7	39.8	
14 years (%)	27.0	20.2	23.1	21.1	
School grade					
7^th^ (%)	45.9	51.4	51.3	47.7	0.81
8^th^ (%)	54.1	48.6	48.7	52.3	
Residence status					
With both parents (%)	88.5	89.0	77.8	80.5	0.10
With one parent and step parent (%)	0.8	0.9	6.0	2.3	
With one parent only (%)	5.7	6.4	10.3	6.3	
With relatives (%)	4.1	3.7	4.3	8.6	
Others (%)	0.8	0	1.7	2.3	

^1^ using chi-squared test of association.

### Effects of the interventions on knowledge

1. Knowledge about health-related aspects of smoking

Scores for knowledge of health-related aspects of smoking are shown in Tables 2 and 3. There was a significant difference between groups in health knowledge scores at baseline (p < 0.001) (Table 2). Following the intervention, there were significant main effects on health knowledge for both the health-based program (β = 3.9 ± 0.6, p < 0.001) and the Islamic-based program (β = 3.8 ± 0.6, p < 0.001) (Table 3). There was also a significant interaction between the health- and Islamic-based programs (β = −3.4 ± 0.9, p < 0.01), indicating that the main effects of the programs were reduced for subjects in the combined (health and Islam) group. There was also some evidence that effects were more similar within each class (ICC = 0.10) (Table 3).

2. Knowledge about Islamic teaching and rulings concerning smoking

**Table 2:** Pre-test comparisons of smoking knowledge, attitude, intentions and behaviors

**Outcomes**	**Health-based (n = 122)**	**Islamic-based (n = 109)**	**Combined (n = 117)**	**Control (n = 128)**	**p values**^**a**^
Health knowledge (mean ± SD)	6.7 ± 2.4***	7.7 ± 2.6	6.8 ± 2.8***	8.6 ± 2.6	< 0.001
Islamic knowledge (mean ± SD)	9.9 ± 3.4***	12.0 ± 2.4	10.1 ± 2.6**	11.4 ± 2.6	< 0.001
Smoking attitude (mean ± SD)	51.1 ± 14.4***	41.5 ± 10.7	46.1 ± 10.1*	42 ± 9.5	< 0.001
Intention to smoke next year, n (%)	28 (23.0)	14 (12.8)	18 (15.4)	17 (13.3)	0.12
Intention to smoke during senior high school, n (%)	32 (26.2)	17 (15.6)	22 (18.8)	19 (14.8)	0.09
Intention to smoke at age 50 or older, n (%)	39 (32.0)	24 (22.0)	24 (20.0)	25 (19.0)	0.08
Past week smoking, n (%)	17 (13.9)	4 (3.7)	4 (3.4)	13 (10.2)	< 0.01
Past month smoking, n (%)	15 (12.3)	4 (3.7)	4 (3.4)	9 (7.0)	< 0.05
Lifetime smoking, n (%)	39 (32.0)	16 (14.7)	18 (15.4)	29 (22.7)	< 0.01

^a^ The comparison was analyzed using ANOVA for knowledge and attitude, and the chi-squared test of association for smoking intentions and behaviors.

* < 0.05, ** < 0.01, *** < 0.001 compared to control group.

**Table 3:** Impact of the health and Islamic-based interventions and their interactions on knowledge and attitude

**Outcomes**	**Main effect of health**				**Main effect of Islam**				**Health × Islam Interaction effect**		**ICC**^**5**^
	**Health (n = 239)**	**Non health (n = 237)**	**β** ± **SE**	**p value**^**1**^	**Islam (n = 226)**	**Non Islam (n = 250)**	**β ± SE**	**p value**^**2**^	**β ± SE**^**3**^	**p value**^**4**^	
Health Knowledge (mean ± SD)											
Visit 1	6.7 ± 2.6	8.2 ± 2.7			7.3 ± 2.7	7.6 ± 2.7					
Visit 2	11.9 ± 2.8	9.8 ± 2.9	3.9 ± 0.6	< 0.001	11.9 ± 2.4	9.8 ± 3.1	3.8 ± 0.6	< 0.001	−3.4 ± 0.9	< 0.01	0.10
Islamic Knowledge (mean ± SD)											
Visit 1	10.0 ± 3.0	11.7 ± 2.5			11.0 ± 2.7	10.7 ± 3.1					
Visit 2	11.5 ± 2.7	12.9 ± 3.0	0.1 ± 0.6	0.88	13.6 ± 2.6	11.0 ± 2.8	3.5 ± 0.5	< 0.001	−2.0 ± 0.8	0.02	0.08
Attitude (mean ± SD)											
Visit 1	48.7 ± 12.7	41.8 ± 10.0			43.9 ± 10.6	46.4 ± 13.0					
Visit 2	42.9 ± 11.0	40.3 ± 10.6	−3.0 ± 1.9	0.14	38.6 ± 10.3	44.3 ± 10.7	−7.1 ± 1.5	< 0.001	5.2 ± 3.0	0.11	0.08

*Note.* Results for the main effects were obtained using a generalized linear model for the outcome with adjustment for baseline values and use of robust standard errors to account for the clustering within both classrooms and schools. In all analyses, the 4 groups were recoded into two factors: (1) Health, and (2) Islamic. Codes of one or zero were used for each factor depending on whether the subjects’ group was one that contained the respective health or Islamic factor. That is, control group (Islamic = 0, Health = 0), Islamic (Islamic = 1, Health = 0), Health (Health = 1, Islamic = 0), combined (Health = 1, Islamic = 1).

^1^ Baseline adjusted effect of Health.

^2^ Baseline adjusted effect of Islam.

^3^The interaction effect represents the additional effect of being in the combined group beyond the separate main effects presented for Health and Islam.

^4^P value for interaction between Health × Islam.

^5^ICC = Intra-class correlations coefficients, from mixed effects random intercept model.

Scores for knowledge of Islamic teaching and rulings concerning tobacco smoking are shown in Tables 2 and 3. There was a significant difference between groups in Islamic knowledge scores at baseline (p < 0.001) (Table 2). Following the intervention, there were significant main effects on Islamic knowledge for the Islamic-based program (β = 3.5 ± 0.5, p < 0.001) but the effects were non-significant for the health-based program (β = 0.1 ± 0.6, p = 0.88). There was a significant interaction between the health- and Islamic-based programs (β = −2.0 ± 0.8, p *=* 0.02), indicating that the main effects of the programs were reduced in those subjects in the combined (health and Islam) group. There was also some evidence that effects were more similar within each class (ICC = 0.08) (Table 3).

### Effects of the interventions on attitude

Scores for attitude toward smoking are presented in Tables 2 and 3. There was a significant difference between groups in attitude scores at baseline (p < 0.001) (Table 2). Following the interventions, there were significant main effects on attitude for the Islamic-based program (β = −7.1 ± 1.5, p < 0.001) but the effects were non-significant for the health-based program (β = −3.0 ± 1.9, p = 0.14) (Table 3). There was a non-significant interaction between the health- and Islamic-based programs (β = 5.2 ± 3.0, p *=* 0.11), suggesting that the main effects of the programs were not significantly different in those subjects in the combined (health and Islam) group. There was also some evidence that effects were more similar within each class (ICC = 0.08) (Table 3).

### Effects of the interventions on intentions to smoke

1. Intention to smoke in next year

The proportions of students who intended to smoke in the next year are presented in Tables 2 and 4. There was no significant difference between groups in intention to smoke in the next year at baseline (p = 0.12) (Table 2). Following the intervention, there were no significant main effects on intention to smoke in next year for either the health-based program (OR = 1.1, 95% CI = 0.3–3.4, p *=* 0.91) or the Islamic-based program (OR = 1.0, 95% CI = 0.3–3.1, p = 0.95) (Table 3). There was limited evidence that the effects were more similar within each class (ICC = 0.04) (Table 4).

2. Intention to smoke during senior high school

**Table 4:** Impact of the health and Islamic-based interventions and their interactions on smoking intentions and behaviors

**Outcomes**	**Main effect of health**				**Main effect of Islam**				**ICC**^**3**^
	**Health (n = 239)**	**Non health (n = 237)**	**OR (95% CI)**	**p value**^**1**^	**Islam (n = 226)**	**Non Islam (n = 250)**	**OR (95% CI)**	**p value**^**2**^	
Intention to smoke next year, n (%)									
Visit 1	46 (19.3)	31 (13.1)			32 (14.2)	45 (18.0)			
Visit 2	27 (11.3)	21 (8.9)	1.1 (0.3,3.4)	0.91	21 (9.3)	27 (10.8)	1.0 (0.3, 3.1)	0.95	0.04
Intention to smoke in senior high school, n (%)									
Visit 1	54 (22.6)	36 (15.2)			39 (17.3)	51 (20.4)			
Visit 2	41 (17.2)	29 (12.2)	1.2 (0.4, 3.2)	0.73	33 (14.6)	37 (14.8)	1.1 (0.4, 3.1)	0.80	0.04
Intention to smoke over 50, n (%)									
Visit 1	63 (26.4)	49 (20.7)			48 (21.2)	64 (25.6)			
Visit 2	55 (23.0)	42 (17.7)	1.2 (0.6, 2.4)	0.52	48 (21.2)	49 (19.6)	1.4 (0.7, 2.7)	0.31	0.03
Past week smoking, n(%)									
Visit 1	21 (8.8)	17 (7.2)			8 (3.5)	30 (12.0)			
Visit 2	2 (0.8)	14 (6.0)	0.1 (0.0,1.5)	0.09	2 (0.9)	14 (5.6)	0.2 (0.0, 2.7)	0.23	0.02
Past month smoking, n(%)									
Visit 1	19 (8.0)	13 (5.5)			8 (3.5)	24 (9.6)			
Visit 2	8 (3.4)	7 (3.0)	0.9 (0.2,4.8)	0.92	7 (3.1)	8 (3.2)	1.9 (0.3, 10.6)	0.45	0.02
Lifetime smoking, n (%)									
Visit 1	57 (23.9)	45 (19.0)			34 (15.0)	68 (27.2)			
Visit 2	54 (22.6)	50 (21.1)	0.7 (0.3,1.6)	0.39	32 (14.2)	72 (28.8)	0.4 (0.2, 1.0)	0.06	0.05

*Note.* Results for the main effects were obtained using a generalized linear model for the outcome with adjustment for baseline values and use of robust standard errors to account for the clustering within both classrooms and schools. In all analyses, the 4 groups were recoded into two factors: (1) Health, and (2) Islamic. Codes of one or zero were used for each factor depending on whether the subjects’ group was one that contained the respective health or Islamic factor. That is, control group (Islamic = 0, Health = 0), Islamic (Islamic = 1, Health = 0), Health (Health = 1, Islamic = 0), combined (Health = 1, Islamic = 1). A Health x Islam interaction term was not included in the models for these outcomes since the main effects for Health and Islam were not significant.

^1^Baseline adjusted effect of Health.

^2^ Baseline adjusted effect of Islam.

^3^ICC = Intra-class correlations coefficients, from mixed effects random intercept model.

The proportions of students who intended to smoke during senior high school are shown in Tables 2 and 4. There was no significant difference between groups in the intention to smoke during senior high school at baseline (p = 0.09) (Table 2). Following the intervention, there were no significant main effects on intention to smoke during senior high school for either the Islamic-based program (OR = 1.1, 95% CI = 0.4–3.1, p = 0.80) or the health-based program (OR = 1.2, 95% CI = 0.4–3.2, p = 0.73) (Table 4). There was limited evidence that the effects were more similar within each class (ICC = 0.04) (Table 4).

3. Intention to smoke when older

The proportions of students who intended to smoke when older are presented in Tables 2 and 4. There were no significant differences between groups in the intention to smoke when older at baseline (p = 0.08) (Table 2). Following the interventions, there were no significant main effects on intention to smoke when older for either the health-based program (OR = 1.2, 95% CI = 0.6–2.4, p = 0.52) or the Islamic-based program (OR = 1.4, 95% CI = 0.7–2.7, p = 0.31) (Table 4). As shown in Table 4, there was little evidence that the effects were less similar within each class (ICC = 0.03) (Table 4).

### Effects of the interventions on smoking behavior

1. Smoking behavior in the past seven days

The proportion of students smoking in the past seven days (past week smoking) is presented in Tables 2 and 4. There was a significant difference between groups in the proportions of past week smoking at baseline (p < 0.01) (Table 2). Following the intervention, there were no significant main effects on past week smoking for either the health-based program (OR = 0.1, 95% CI = 0.0–1.5, p = 0.09) or the Islamic-based program (OR = 0.2, 95% CI = 0.0–2.7, p = 0.23) (Table 4). There was little evidence that the effects were more similar within each class (ICC = 0.02) (Table 4).

2. Smoking behavior in the past 30 days

The proportion of students smoking in the past 30 days (past month smoking) is presented in Tables 2 and 4. There was a significant difference between groups in the proportions of past month smoking at baseline (p < 0.05) (Table 2). Following the intervention, there were no significant main effects on past month smoking for either the health-based program (OR = 0.9, 95% CI = 0.2–4.8, p = 0.92) or the Islamic-based program (OR = 1.9, 95% CI = 0.3–10.6, p = 0.45) (Table 4). There was limited evidence that effects were more similar within each class (ICC = 0.02) (Table 4).

3. Smoking behavior in lifetime

The proportion of students that had smoked in their lifetime is shown in Tables 2 and 4. There was a significant difference between groups in lifetime smoking behaviors at baseline (p < 0.01) (Table 2). Following the intervention, there were no significant main effects on lifetime smoking behaviors for either the health-based program (OR = 0.7, 95% CI = 0.3–1.6, p = 0.39) or the Islamic-based program (OR = 0.4, 95% CI = 0.2–1.0, p = 0.06) (Table 4). There was also some evidence that effects were more similar within each class (ICC = 0.05) (Table 4).

## Discussion

This study investigated the impact of school-based smoking prevention programs on adolescents’ smoking knowledge, attitude, intention and behaviors in Aceh, Indonesia. The primary objective of the study was to identify appropriate strategies for smoking prevention programs among Indonesians adolescents, especially in Aceh. It was anticipated that findings of this study would help provide practical recommendations for professionals within Indonesia and perhaps other Muslim populations to challenge the high rates of smoking prevalence, morbidity, and mortality across the country.

Findings from this study suggest that the school-based programs were effective in increasing students’ knowledge about both health-related aspects of cigarette smoking and Islamic teaching and rulings on tobacco smoking in Aceh. Knowledge about health-related aspects of tobacco smoking increased among participants in all of the intervention programs. However, knowledge about Islamic teaching and rulings on smoking increased only in the Islamic-based and combined programs. One possible explanation for this is that participants in the Islamic-based program received a large amount of information about Islamic teaching and the health-related effects of tobacco smoking. Participants in the combined program received components of both the health- and Islamic-based programs, while participants in the health-based program received no information about tobacco smoking from an Islamic viewpoint.

Our results extend findings from previous studies and reviews of school-based smoking prevention programs among adolescents in non-Muslim countries that found school-based programs had a positive impact on adolescents’ smoking-related knowledge [9,10,39,40]. A previous meta-analysis of 47 school-based smoking programs conducted by Rundall and Bruvold [41] showed that 98% of the reviewed programs successfully increased participants’ knowledge in treatment groups. Similarly, a more recent meta-analysis [8] showed that 73% of 11 school-based smoking prevention programs resulted in significant improvement in participants’ knowledge about smoking.

Attitude towards smoking of participants in the Islamic-based intervention group improved significantly following the intervention. This finding is consistent with previous work in school-based smoking prevention programs reported in other areas of the world. In Greece, a school-based peer-led smoking prevention program improved anti-smoking attitude [23], and in Yilan County, Taiwan [9] a significant change in anti-smoking attitude was observed among junior high school students after a one-week program intervention. In their review of 11 school-based smoking prevention programs in South Korea, Park [8] reported small, medium, and large effects for four, three, and two studies respectively.

Our study demonstrates that Islamic teaching has a significant role to play in increasing anti-smoking attitude among students in Indonesia. Our analysis suggests that the attitude changes were larger among participants in the Islamic-based program compared with non-Islamic programs. These findings support the recommendation of the educators in our preliminary feasibility test which suggested school-based smoking prevention programs in Aceh would be most effective if they included Islamic teaching and rulings concerning tobacco smoking.

Having an intention to smoke is considered an important predictor of smoking behaviors [33,37]. In a review of previously published reviews of school-based tobacco use prevention programs by Dobbins et al. [42] one of two reviews reported positive effects of the programs in reducing smoking intentions while the other reported a promising effect. In our study we also found a positive effect of the programs on the smoking intention, although the effects were insignificant and similar for both the health- and Islamic-based program.

In a previous review [42], only six of 12 reviews found positive effects of tobacco use prevention programs on smoking behaviors, with two reporting promising effects, and three no effects. Park [8] found no significant effects of school-based programs on smoking behaviors. Our findings were consistent with these studies showing only insignificant reductions in smoking behaviors for both the health- and Islamic-based programs. This was not however unexpected given the relatively low prevalence of smoking amongst the participants.

The ICCs for outcome variables were generally small to moderate, with the highest being for health knowledge (0.10) and the lowest for the weekly and monthly smoking variables (0.02). Overall, the ICCs for knowledge and attitude (continuous variables) were slightly higher than the ICCS for the smoking behaviors and intentions (categorical variables). This finding suggests that the effects of the program on participants within the same class were more highly correlated with respect to their knowledge and attitude when compared to the effects on behavior and intentions.

There are some potential limitations in this study. One of the limitations of this study is the differences between groups at baseline in knowledge, attitude, and behaviors after randomization due to the cluster-randomized nature of the study with randomization of schools rather than individuals. Although we adjusted for the differences in these variables at baseline in our models, there may still have been residual confounding. For example, students with lower levels of knowledge may also have been different in other characteristics which could have led to the intervention being less effective in this group. In addition, the low smoking rates in certain behavior and intention categories imposed constraints on estimating each program’s effectiveness for behaviors. Additionally, outcome measures were assessed using a self-reported questionnaire and participants might be inclined to underestimate their tobacco use. Although it would have been possible to validate students’ responses about their smoking behaviors using biochemical tests, we were unable to do so because the study was limited by the number of personnel, and technical and financial constraints. The questionnaire also relied on students’ recall ability over long time periods and the limitations of these were evident in their responses between baseline and follow-up questionnaires regarding their lifetime use of tobacco. Validation of our results with other instruments and biochemical tests would therefore be useful. Finally, this study only reported on the short-term impacts of the program; longer-term evaluation of the program is required to determine if the effectiveness of the intervention is sustainable in the longer term.

Notwithstanding these limitations, this study is the first RCT to assess the effectiveness of school-based smoking prevention programs in Indonesia, using specifically developed interventions that included health- and Islamic-based concepts.

## Conclusions

This study may have several implications for smoking prevention programs among Indonesian adolescents and those in other Muslim countries. The study demonstrated that school-based smoking prevention programs increase students’ knowledge of smoking and its harmful effects, and elicit a better anti-smoking attitude. The study also suggests that either an Islamic or Health-based program is suitable for students in Aceh and perhaps other Muslim societies. Combining the 2 programs does not however lead to greater effectiveness. Finally, we recommend further research to replicate this program intervention approach with a more rigorous study design that ensures better balance at baseline, populations with a higher prevalence of smoking, and longer term programs and evaluation so that the findings of this study and the long-term effectiveness of the programs can be established.

## Competing interests

All authors have no competing interest.

## Authors’ contributions

All authors participated in designing the study, implementing program interventions, conducting program evaluations, and the writing of the manuscript. Specifically, TT designed the concept of the study, was the main author of the manuscript, coordinated the program implementation, administration and testing of students, and completed data analysis; PRW participated in the development of the grant proposal, program protocol and manuscript preparation; JC contributed in program development and design and manuscript preparation; and RJW contributed in grant writing, data analysis and manuscript preparation. The final manuscript was read and approved by all authors.

## Pre-publication history

The pre-publication history for this paper can be accessed here:

http://www.biomedcentral.com/1471-2458/13/367/prepub
